# Low-Temperature Curable Negative-Tone Photosensitive Polyimides: Structure and Properties

**DOI:** 10.3390/polym15040973

**Published:** 2023-02-16

**Authors:** Sheng-nan Fan, Li-li Yuan, Li-zhe Wang, Bin Jia, Jia-xin Ma, Hai-xia Yang, Shi-yong Yang

**Affiliations:** 1 Key Laboratory of Science and Technology on High-tech Polymer Materials, Institute of Chemistry, Chinese Academy of Sciences, Beijing 100190, China; 2 School of Chemical Sciences, University of Chinese Academy of Sciences, Beijing 100049, China; 3 Minseoa Advanced Polyimide Corporation, Beijing 101300, China

**Keywords:** photosensitive polyimide, low-temperature imidization, photo-patterning, mechanical properties

## Abstract

Low-temperature curable negative-tone photosensitive polyimide (n-LTPI) viscous solutions were prepared by dissolving photo-crosslinkable poly (amic ester) (pc-PAE) resin, photophotocrosslinker, photoinitiator, and the heteroaromatic base as curing catalysts, and other additives in organic solvents. Among them, the pc-PAE resin was synthesized by polycondensation of aromatic diacid chloride and diester of 2-ethoxymathacrylate, aromatic diamines in aprotic solvents. After being spun-coated on a silicon wafer surface, soft-baked, exposed to UV light, and developed, the n-LTPI with 2% of imidazole (IMZ) as a curing catalyst produced high-quality photo-patterns with line via resolution of 5 μm at 5 μm film thickness. The photo-patterned polymer films thermally cured at 230 °C/2 h in nitrogen showed 100% of the imidization degree (ID) determined by in situ FT-IR spectroscopy. The thermally cured polymer films exhibited great combined mechanical and thermal properties, including mechanical properties with tensile strength of as high as 189.0 MPa, tensile modulus of 3.7 GP, and elongation at breakage of 59.2%, as well as glass transition temperature of 282.0 °C, showing great potential in advanced microelectronic packaging applications.

## 1. Introduction

Photosensitive polyimides (PSPI) have been widely employed as passivation layers or interlayer dielectrics of multilayer structures in microelectronic manufacturing and advanced packaging due to their excellent combined properties, including great photo patterning performance, high strength and toughness, outstanding thermal stability and chemical resistance, etc. [1,2,3,4]. PSPIs could be divided into negative-tone and positive-tone according to the photolithographic performance, and can also be divided into poly(amic acid) (PAA)-type and poly(amide ester) (PAE)-type according to the precursor. The first practical negative-tone PSPI(n-PSPI), reported by Rubner in 1976 [5], was prepared by dissolving the PAE with acryloyl groups in the polymer backbone and the photo-packages in organic solvent, which showed the long storage stability and good photosensitivity. Since then, great progress on the improvements of n-PSPI performance has been achieved [6,7,8]. Recently, the advanced packaging technology, such as fan-out wafer-level packaging (FO-WLP), 2.5/3D integration, and packaging, required that n-PSPI could be thermally cured at a lower temperature (<250 °C) [9,10,11,12]. However, the conventional thermal imidization of PAE into polyimide must be completed at a higher temperature (300–350 °C) [13,14,15,16].

Many papers have been published on the imidization of PAA and PAE [17,18,19]. Due to the interaction between the carboxyl group in the PAA molecule and the aprotic solvent, the curing temperature of PAA is usually lower than that of PAE with the same main chain structure [20]. For example, Tae Joo Shin et al. [21] selected PAE (PMDA-ODA) as the model, which began to undergo imidization at 216 °C, and the ID reached 97% at 356 °C. However, PAA(PMDA-ODA) starts to convert at 124 °C and is finished at 310 °C. It was found that benzimidazole, a heteroaromatic base, could catalyze the thermal imidization of PAA into polyimide at 100 °C/24 h [22]. 1,8-diazabicyclo [5.4.0] undec-7-ene (DBU) was also effective to reduce the imidization temperature of PAA to 200 °C [23,24]. Huang C. et al. [25] have investigated the mechanical, thermal, and electrical properties of the polyimide films derived from the imidization of PAA catalyzed by quinoline (QL) and quinoline derivatives. Sui, Y.Y. [26] found that the curing temperature of PAA could be reduced to 200 °C catalyzed by 5-aminobenzimidazole as an active curing catalyst. Although the imidization of PAA can be catalyzed by heteroaromatic bases, there are few reports on the imidization of PAE, which is usually used in n-PSPIs [27,28,29]. The experimental results of Li W. S. et al. [30] indicated that the imidization reaction of PAE could start at around 100 °C and complete at 270 °C, which was much lower than the previously reported temperature [31]. Frank Windrich et al. [32] studied an PAE-type n-PSPI purchased by Fujifilm Electronic Materials (LTC9300 series) and found that the imidization of non-crosslinked PAE was completed at 250 °C. By contrast, UV-exposed PAE required raising the temperature to above 340 °C to achieve fully imidized polymer films.

In this study, n-PSPIs were prepared by dissolving the photo-crosslinkable poly (amic ester) (pc-PAE) resin, photo-photocrosslinker, photoinitiator, and heteroaromatic base as curing catalysts in organic solvents. The impacts of the polymer backbone structures of pc-PAE, as well as the molecular structures of heteroaromatic base catalysts on n-PSPI photo-patterning performance, have been systematically investigated. The n-PSPI with heteroaromatic base as a curing catalyst showed good photo-patterning performance. The photo-patterned polymer films could be thermally cured at 230 °C/2 h in nitrogen, giving the completed imidized polymer films great combined mechanical and thermal properties.

## 2. Experimental

### 2.1. Materials

4,4′-oxydianidine (ODA) and 3,3′,4,4′-biphenyltetracarboxylic dianhydride (BPDA) were purchased from China-tech (Tianjin, China) Chemical Co., Ltd. Dimethylacetamide (DMAc) and N-methyl-2-pyrrolidinone (NMP) was purchased from Concord Technology (Tianjin, China) Co., Ltd. and used as received. 2-Hydroxyethyl methacrylate (HEMA) and thionyl chloride (SOCl_2_) were purchased from Aladdin Bio-Chem Technology (Shanghai, China) Co., Ltd. Other commercially available solvents and reagents were purchased from InnoChem Sci. Technol. (Beijing, China) Co. Ltd. and used as received.

### 2.2. Synthesis of pc-PAE Resin

52.06 g (0.40 mol) of 2-hydroxyethyl methacrylate (HEMA), 28.44 g (0.36 mol) of pyridine, 0.22 g (0.002 mol) of hydroquinone (HQ), and 58.84 g (0.20 mol) of BPDA were dissolved in 250 g of NMP in a 500 mL three-necked round bottom flask equipped with a mechanical stirrer, a thermometer, and a nitrogen inlet. The solution was heated to 50 °C and stirred for 6 h. Then, the reaction mixture was cooled to 0–10 °C by using an ice-bath, and 43.60 g (0.36 mol) of SOCl_2_ was added slowly by using an addition funnel. The ice-bath was removed after 2 h, and was then stirred at room temperature for 4 h. The solution was cooled to below 10 °C, and then 36.06 g (0.18 mol) of ODA and 280 g of NMP were added. The reaction mixture was stirred at room temperature for 10 h. The resulting viscous polymer solution was poured into deionized water (5 L) with a thin stream to yield silky resin. The precipitate was collected and dried at 50 ^◦^C under vacuum for 24 h to afford a pc-PAE resin. Yield: 122.9 g (86.0%).

1H-NMR(DMSO-d6): δ(ppm)=1.87 (-CH_3_); 4.32 and 4.50 (-OCH_2_CH_2_O-); 5.62 and 6.00 (=CH_2_); 7.02–8.26 (Ar-H);10.55 (-CONH-). The number average molecular weight (Mn), weight average molecular weight (Mw), and polydispersity of PAE-1 were Mn = 11,562, Mw = 21,150, and PDI = 1.83, respectively.

### 2.3. Preparation of Low-Temperature Curable Photosensitive Polyimides (n-LTPIs)

In a clean room with yellow light, 50.00 g of pc-PAE resin was dissolved in 80.00 g of NMP to give a homogeneous solution in a 500 mL three-necked round bottom flask equipped with a mechanical stirrer, a thermometer, and a nitrogen inlet, to which 3.00 g of 1,2-ethanediyl bis (2-methylacrylate)-ethylene as photocrosslinker, 0.500 g of 2-benzyl-2-(dimethylamino)-4′-morpholinobutyrophenone as photoinitiator, 1.00 g of phenylbis (2,4,6-trimethylbenzoyl) phosphine oxide, and 1.00 g of imidazole (IMZ) as a curing catalyst were added, successively, and dissolved with stirring in nitrogen to give n-LTPI-IMZ-2.0 viscous solution with viscosity of 3500–3700 mPa.s at 35% of solid content, which was then filtrated through a 0.20 μm Capsule filter and stored in −18 °C.

Similarly, a series of n-LTPIs with different heteroaromatic base as curing catalysts were prepared by a similar method to n-LTPI-IMZ-2.0, except that the based catalyst was replaced by quinoline (QL) (n-LTPI-QL-2.0), isoquinoline (IQL) (n-LTPI-IQL-2.0), 2,6-Dimethylpiperidine (DMP) (n-LTPI-DMP-2.0), and 1H-benzimidazole (BZI) (n-LTPI-BZI-2.0), respectively.

In comparison, a photosensitive polyimide without the addition of any heteroaromatic base as curing catalyst (n-LTPI-0) was also prepared.

### 2.4. Measurements

1H NMR spectra were recorded on a Bruker Avance 400 Spectrometer (Billerica, MA, USA) in CDCl3 or DMSO-d6. The number-average molecular weights (Mn), the weight-average molecular weights (Mw), and the polydispersity indices (PDI) were measured by a gel permeation chromatography (GPC) system (Waters e2695, America) using NMP containing 0.02 M H_3_PO_4_ as eluent at a flow rate of 0.7 mL/min at 50 °C. Solubility was measured by dissolving 1.0 g of pc-PAE in 9.0 g of organic solvents (10 wt.% concentration) and was stirred for 24 h at room temperature.

The film thickness was measured by a Nano Spec II full-automatic film thickness tester. The lithographic performance was investigated by an optical microscope (MX63, Olympus Corporation, Tokyo, Japan) and a scanning electronic microscope (SEM, s-9380, Hitachi High-Tech Corporation, Tokyo, Japan). Mechanical properties were tested on an Instron-3365 tensile apparatus. The glass transition temperature (T_g_) and mechanical properties at high temperatures were analyzed by a Dynamic Mechanical Analysis (DMA, TA Q800, TA instruments, New Castle, DE, USA) instrument under nitrogen. The storage module-temperature curve was obtained at a heating rate of 5 °C/min, and the stress–strain curve at a specific temperature was obtained at a rate of 0.1 N/s. Thermal stability was analyzed by a Thermogravimetric Analyzer (TGA, TA Q50, TA instruments, New Castle, DE, USA) instrument at a heating rate of 20 °C/min under nitrogen. Electrical properties were measured on a Vector network analyzer (N5227B PNA, Keysight Technologies, Santa Rosa, CA, USA) in 10 GHz.

### 2.5. Imidization Degree

Imidization reaction is the fractional conversion of the ester groups to imides. FT-IR transmission spectra were recorded on a spectrometer (tensor 27, Bruker, Billerica, MA, USA) equipped with a vacuum heated cell, which has the function of in situ rapid-scan. The n-LTPI sample was spin-coated on potassium bromide (KBr) crystal discs, heated at 100 °C for 10 min to remove the excess solvent, and a film with a thickness of about 0.5 μm was obtained on the KBr surface. The spectra changes were followed over time measuring the imide absorption at 1370 cm^−1^ (axial ν(C-N-C)), normalized to the aromatic ring band at 1500 cm^−1^ (ν(C_6_H_4_)) [30]. The imidization degree (ID) was then calculated by the following equation [33],

(1) $$\mathrm{Degree}\;\mathrm{of}\;\mathrm{Imidization}\;\left(\mathrm{ID}\right)=\frac{h_{\mathrm{sample}}\left({1380\;\mathrm{cm}}^{-1}\right){/h}_{\mathrm{sample}}\left({1500\;\mathrm{cm}}^{-1}\right)}{h_{\mathrm{cured}}\left({1380\;\mathrm{cm}}^{-1}\right){/h}_{\mathrm{cured}}\left({1500\;\mathrm{cm}}^{-1}\right)}\times 100\%$$

Subscript “sample” after the ratio refers to the polymer baked at each temperature for 2 h. “Curing” refers to the sample after complete curing, and here it refers to the sample after curing at 350 °C for 2 h.

### 2.6. Photo-Patterning of n-LTPIs

The n-LTPI viscous solution was spin-coated on a 12-inch silicon wafer at 3000 rpm/30 s, and then soft-baked at 110 °C/4 min on a Coater/Developer (CLEAN TRACK™ ACT™ 12, Tokyo Electron Device LTD., Tokyo, Japan). After being exposed on an i-line stepper (FPA-5550iZ2, Canon Inc. Operations, Tokyo, Japan) at 600 mJ/cm^2^, the photo-pattern on wafer was developed with cyclopentanone. The photo-patterned polymer coating was thermally cured at 230 °C/2 h in an oxygen-free oven (O_2_ < 100 ppm). The thermally cured polyimide films with 10 μm thickness for mechanical and thermal property testing were separated from the wafer by soaking in a dilute solution of hydrofluoric acid.

## 3. Results and Discussion

### 3.1. Synthesis and Characterization

**Scheme 1:**
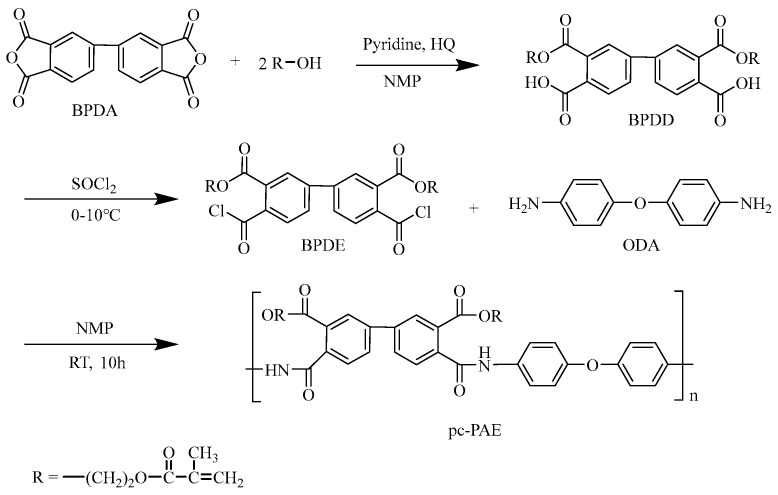
Synthesis of photo-crosslinkable poly (amic ester).

The photo-crosslinkable poly(amic ester) (pc-PAE) resin can be photo-crosslinked by UV light exposed (i or g lines) due to the side groups of 2-ethoxymathacrylate on the polymer backbones. It was synthesized by polycondensation of aromatic diacid chloride and diester of 2-ethoxymathacrylate (BPDE) and aromatic diamine (ODA) in aprotic solvents (Scheme 1). BPDE was prepared by esterification of BPDA with 2-hydroxyethyl methacrylate (HEMA) in the presence of pyridine and hydroquinone (HQ) in NMP as a solvent to give an aromatic diacid and diester of 2-ethoxymathacrylate (BPDD), followed by treatment with thionyl chloride (SOCl_2_) to yield the corresponding aromatic diacid chloride and diester (BPDE) at low temperatures (0–10 °C).

**Figure 1:**
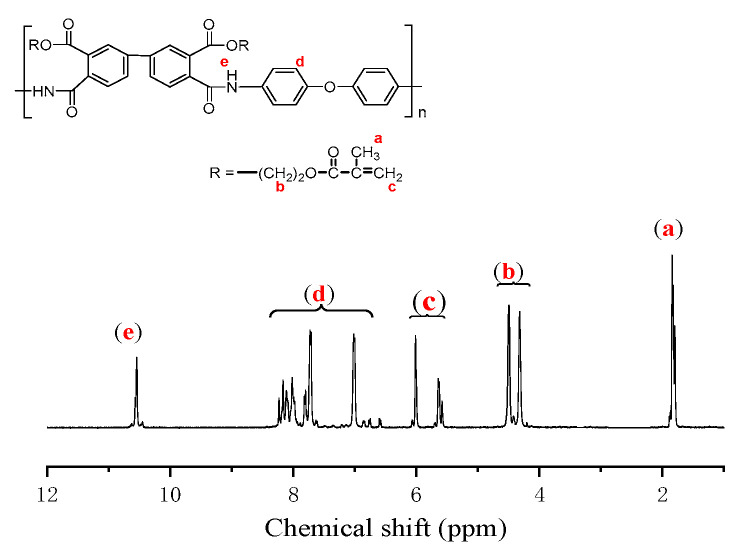
^1^H NMR spectrum of the pc-PAE resin.

Figure 1 depicts the ^1^H NMR spectrum of the pc-PAE resin. The absorptions at 10.55 ppm (e) and 7.02–8.26 ppm (d) were designed as the protons of amide groups (-CONH-) and of aromatic phenyl groups (Ar-H) in the polymer backbone, respectively. The peaks at 1.87 ppm (a), 4.32–4.50 ppm (b), and 5.62–6.00 ppm (c) were attributed to the protons of -CH_3_, -OCH_2_CH_2_O-, and =CH_2_ of ethoxylmathacrylate side groups (-O-CH_2_CH_2_-O-C(O)-C(CH_3_)=CH_2_) in the polymer backbones, indicating that the pc-PAE has the expected chemical structure. The number average molecular weight (Mn), weight average molecular weight (Mw), and polydispersity of the pc-PAE resins were measured by GPC in the range of 1.2 × 10^4^, 2.1 × 10^4^, and 1.83, respectively, corresponding the polymerization degree of 29–30.

**Figure 2:**
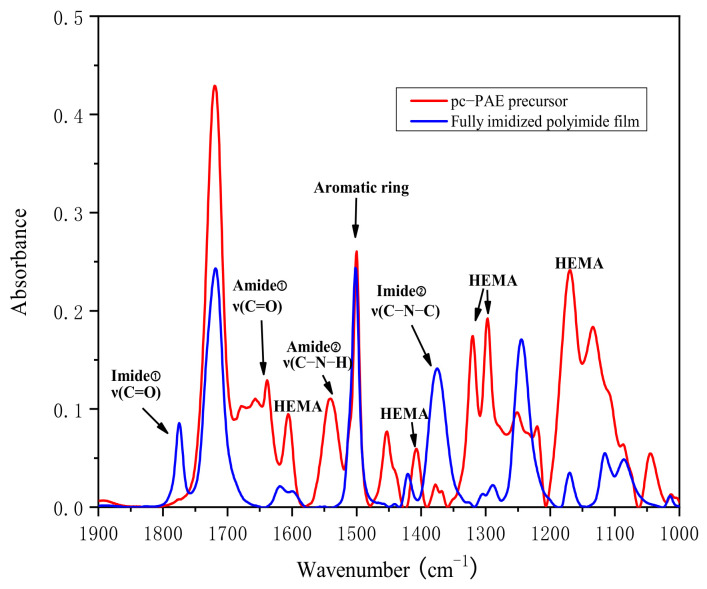
FT-IR spectra of the pc-PAE resin (red) and the fully imidized polymer films (blue).

Figure 2 compares the FT-IR spectra of the pc-PAE resin and the thermally cured polyimide films. The pc-PAE resin showed characteristic absorptions of aromatic amide groups at 1720 cm^−1^(C=O, stretching vibration), 1640 cm^−1^(C=O, stretching), 1550 cm^−1^(C-N, streching), and the absorptions of aromatic phenylene at 1600–1650 cm^−1^(C-H, stretching vibration) and 1500 cm^−1^(C-C, stretching vibration). After being thermally cured at 350 °C/2 h, the fully imidized polymer film showed the characteristic peaks of the imide groups at 1780, 1720, and 1370 cm^−1^. However, the characteristic peaks of HEMA in the pc-PAE resin at 1604, 1404, 1320, 1290, and 1170 cm^−1^ disappeared [32], indicating that the photo-crosslinkable poly (amic resin) resin was completely converted into polyimide after being thermally cured at 350 °C/2 h.

### 3.2. Photo-Patterning Performance

**Figure 3:**
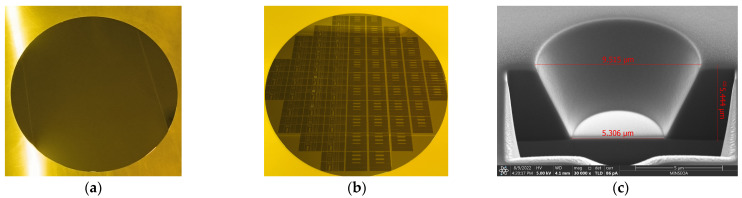
Photo-patterning of n-LTPI: (**a**) Spin-coated and soft-baked coating on 12-inch wafers. (**b**) The photo-patterning on wafer. (**c**) With 5 μm diameter.

The n-LTPI solution was spin-coated on a 12-inch silicon wafer at 1500–3000 rpm to give a coating with 9–10 μm thickness, and then soft-baked at 110 °C/4 min. on a Coater/Developer Track (Figure 3a). After being exposed under UV light (i line) on a stepper at 300–800 mJ/cm^2^, and being developed with cyclopentanone, the photo-patterned polymer coating was obtained (Figure 3b,c), which was then thermally cured at 230 °C/2 h in an oxygen-free oven (O_2_ < 100 ppm) to give the thermally cured polyimide films with 3–10 μm thickness. The n-LTPIs showed high-quality photo-patterning performance via a resolution of 5–10 μm at 5–10 μm film thickness (Table 1). The film retention rates after development were 85%~92%. The introduction of heteroaromatic bases had no negative impact on the photolithography of n-LTPI. Among them, the resolution of n-LTPIs with DMP and IMZ was significantly improved, and the film retention rate was decreased. It showed that the addition of DMP and IMZ improved the solubility of n-LTPI, which was within the acceptable range.

### 3.3. Low Temperature Imidization

**Figure 4:**
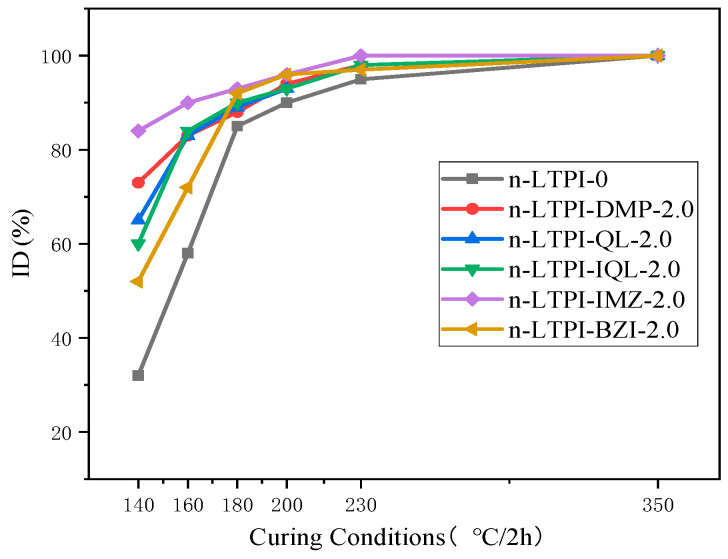
Effect of curing temperature on imidization degree (ID) of the thermally cured films with different heteroaromatic bases.

**Figure 5:**
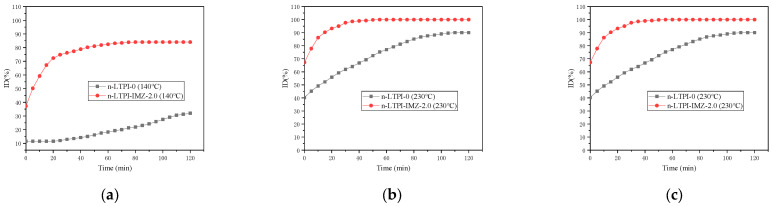
Effect of curing time on imidization degree (ID) of the polymer films with 2.0% of IMZ at different curing temperatures: (**a**) 140/2 h, (**b**) 230/2 h, (**c**) 350/2 h.

Figure 4 compares the effect of the curing temperature on imidization degree of the thermally cured polymer films with different heteroaromatic bases. The heteroaromatic bases employed as curing catalysts are 2,6-Dimethylpiperidine (DMP, b.p. 127.1 °C), quinoline (QL, b.p. 237.7 °C), isoquinoline (IQL, b.p. 243.2 °C), imidazole (IMZ, b.p. 257.0 °C), and 1H-benzimidazole (BZI, b.p. 360.0 °C). Compared with n-LTPI-0 without a heteroaromatic base as a curing catalyst, n-LTPIs with 2 wt.% of heteroaromatic bases exhibited higher ID values at lower curing temperatures (230 °C/2 h). n-LTPI with 2.0% of IMZ (n-LTPI-IMZ-2.0) showed an ID value of 90% after being thermally cured at 160 °C/2 h, much higher than that of other heteroaromatic bases (DMP/QL/IQL, ID ≈ 80%, and BZI, ID = 72%). Figure 5 compares the impacts of the curing time on ID values of the polymer films with 2.0% of IMZ cured at different curing temperatures determined by in situ FT-IR. At a lower curing temperature (140 °C, Figure 5a), the ID values of the thermally cured polymer films could only reach 82% after the curing time was longer than 60 min. At 230 and 350 °C (Figure 5b,c), the ID value reached 100% after the curing time was over 40 min. and 20 min., respectively. The results showed that the heteroaromatic base catalyst, which has a significant catalytic effect on the imidization of PAA, also has a significant effect on PAE. Therefore, the 230 °C curable negative-tone PSPI could be prepared by the addition of 2% IMZ as a curing catalyst.

**Scheme 2:**
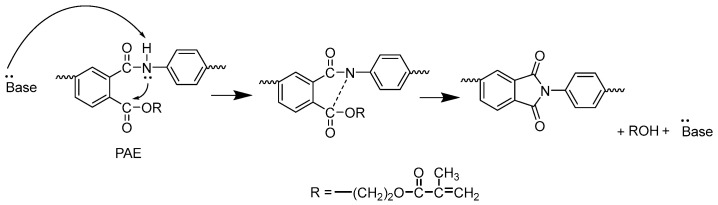
Mechanism of the low-temperature imidization of PAE catalyzed by heteroaromatic bases.

The conventional negative-tone photosensitive polyimide derived from the pc-PAE resin showed poor film-forming ability after being cured at a low temperature (<250 °C/2 h). For instance, the 230 °C/2 h cured polymer films usually showed low glass transition temperature and mechanical strength and toughness at elevated temperature, which cannot meet the advanced packaging application requirements. The heteroaromatic base was found to be an effective catalyst for the imidization reaction of pc-PAE resins with the catalytic mechanism, as shown in Scheme 2 [21]. The lone pair electrons of the heteroaromatic base molecule could first attack the hydrogen atoms of the amide groups, promoting the lone pair electrons of the nitrogen atoms in the amide group to attack the carbon atoms of the ester group, and resulting in the bond breakage of the ester group. Then, the ring-closure reaction of imide was accomplished by evolving aliphatic alcohol as a byproduct derived from the R groups in the pc-PAE [17].

### 3.4. Mechanical Properties

Table 2 compares the mechanical properties of the thermally cured n-LTPI films at 230 °C/2 h with different heteroaromatic bases as curing catalysts. The addition of heteroaromatic bases into n-LTPIs showed obvious impacts on the mechanical properties of the 230 °C/2 h cured polymer films. Compared to the thermally cured n-LTPI film without the addition of heteroaromatic base (n-LTPI-0), the n-LTPIs with 2.0% of QL or IQL (i.e., n-LTPI-QL-2.0 and n-LTPI-IQL-2.0) showed obvious lower mechanical strength and toughness. For instance, n-LTPI-QL-2.0 showed tensile strength of 113.4 MPa and elongation at breakage of 19.2%, respectively, lower than that of n-LTPI-0 (180.7 MPa and 59.1%). However, the n-LTPIs with 2.0% of IMZ (n-LTPI-IMZ-2.00) exhibited tensile strength of 189.0 MPa and elongation at breakage of 59.2%, identical to n-LTPI-0. Meanwhile, the ID value of the 230 °C/2 h cured polymer films was measured at 100.0% by FT-IR spectrometry, better than n-LTPI-0 (95.1%). It was found that the loadings of heteroaromatic bases in n-LTPIs also have apparent impact on the mechanical properties of the 230 °C/2 h cured n-LTPI films (Table 3). The ID values reached 100% when the IMZ loading was over 2.0%. The thermally cured films of n-LTPI with 1.0% of IMZ (n-LTPI-IMZ-1.0) showed the highest tensile strength (190.2 MPa) and elongation at breakage (61.2%), and the ID value was 99.2%. However, when the loading of IMZ is over 5.0%, the mechanical strength and toughness were reduced gradually. For instance, the thermally cured films of n-LTPI with 5.0% of IMZ(n-LTPI-IMZ-5.0) showed tensile strength of 156.8 MPa and elongation at breakage of 36.1%, respectively, being obviously reduced compared to n-LTPI-0.

### 3.5. Thermal and Electrical Properties

**Figure 6:**
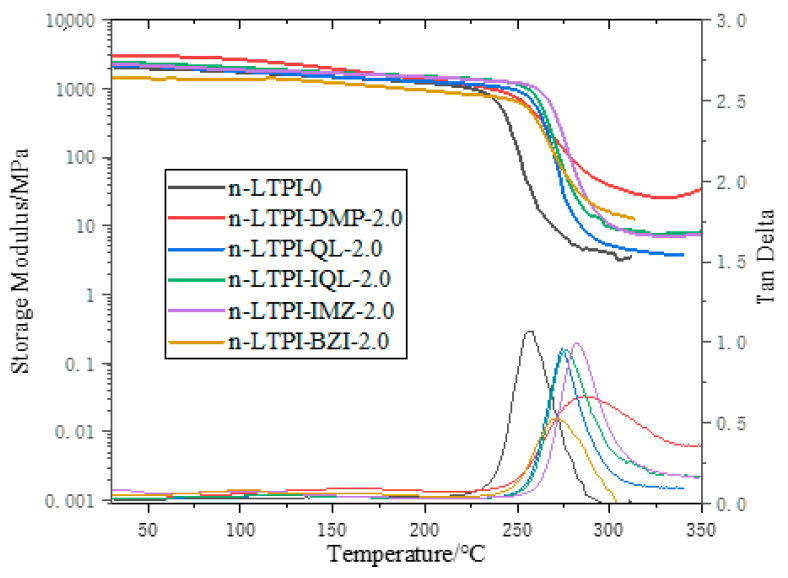
DMA curves of the 230 °C/2 h cured n-LTPI films with different heteroaromatic bases.

**Figure 7:**
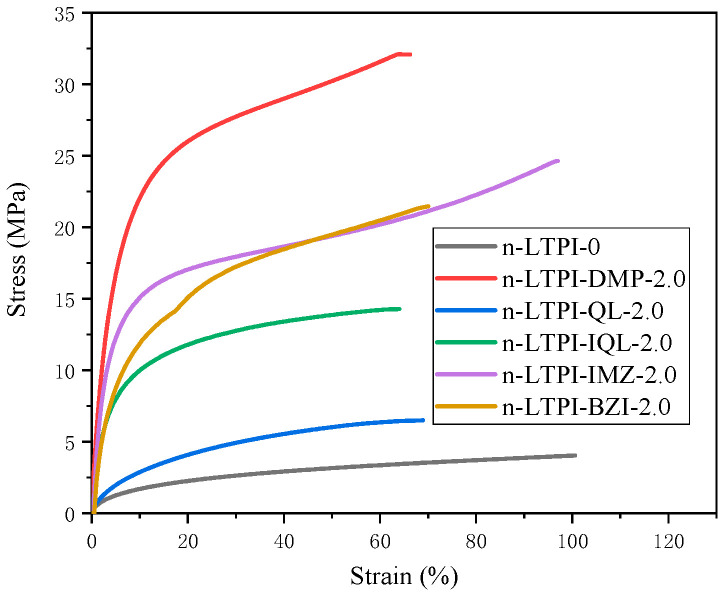
DMA stress–strain curves at 245 °C of the thermally cured n-LTPI films with different heteroaromatic bases.

Figure 6 compares the DMA curves of the 230 °C/2 h cured n-LTPI films with different heteroaromatic bases. Compared with n-LTPI-0 without any curing catalyst, the n-LTPIs with 2.0% loading of heteroaromatic base as curing catalysts showed improved thermal properties. The glass transition temperatures were increased by 15–30 °C compared to that of n-LTPI-0, of which n-LTPI-IMZ-2.0 showed the highest T_g_ value of 280 ^◦^C. Figure 7 depicts the DMA stress–strain curves at 245 °C of the 230 °C/2 h cured n-LTPI films with different heteroaromatic bases. Although the thermally cured n-LTPI-0 showed the highest strain (100%), its stress is very low (<5 MPa). However, the n-LTPI- IMZ-2.0 and n-LTPI-DMP-2.0 both showed higher mechanical strengths (>25 MPa) and toughness (>60%) at high temperatures (245 °C). For instance, the stress and strain of n-LTPI-IMZ-2.0 at 245 °C were measured at 24.7 MPa and 96.8%, respectively.

**Figure 8:**
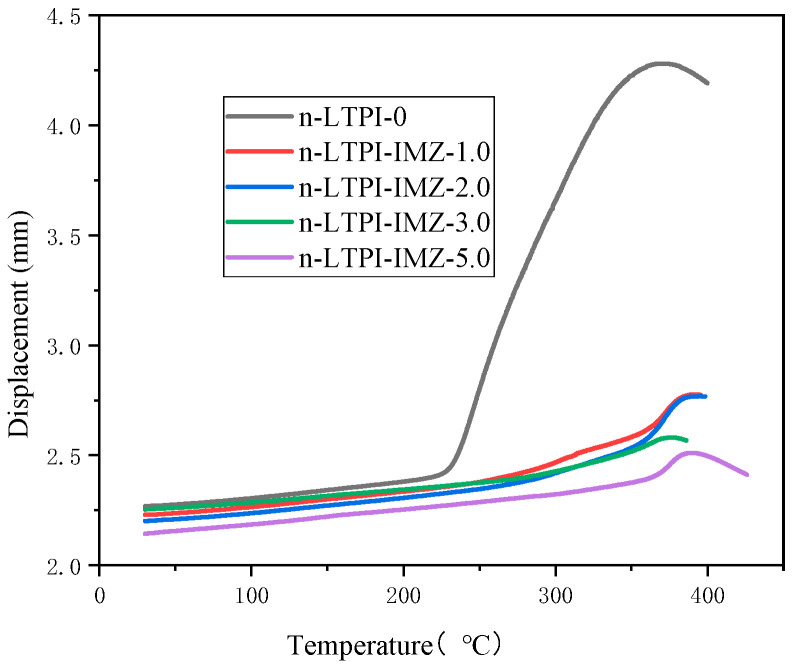
TMA of the 230 °C/2 h cured n-LTPI films with different IMZ loadings.

**Figure 9:**
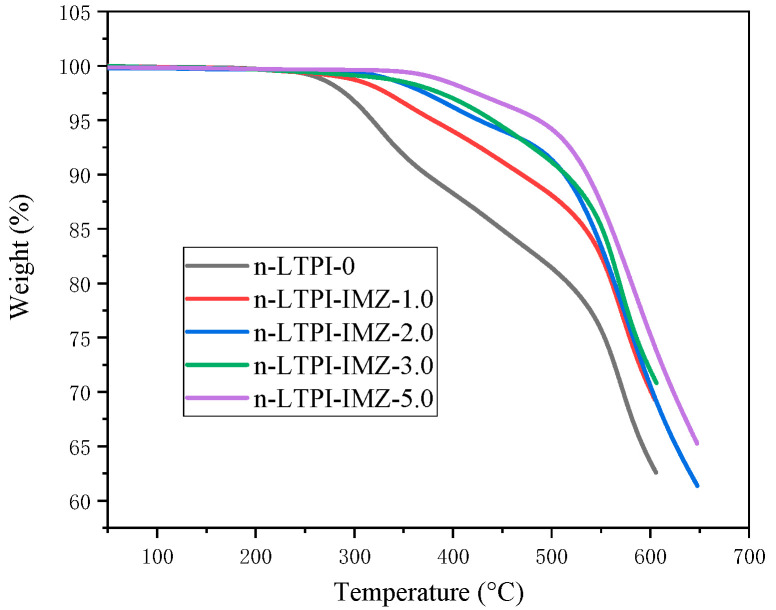
TGA of the 230 °C/2 h cured n-LTPI films with different IMZ loadings.

Table 4 shows thermal and electrical properties of the thermally cured n-LTPI films at 230 °C/2 h with different loadings of heteroaromatic bases as curing catalysts. Compared with n-LTPI-0, n-LTPIs with different IMZ loadings (1.0% to 5.0 wt.%) showed improved thermal stability, including a T_g_ values increase of 16.8–38.7 °C and a T_5_ values increase of 55.1–173.8 °C, respectively. Figure 8 compares the TMA curves of the 230 °C/2 h cured n-LTPI films with different IMZ loadings. The Coefficient of Thermal Expansion (CTE) of the 230 °C/2 h cured n-LTPI-0 film was measured at 52.4 ppm/°C at 50–200 °C, slightly higher than 47.3–49.2 ppm/°C of the cured n-LTPIs films with different IMZ loadings. By increasing IMZ loadings from 1.0 wt.% to 5.0 wt.%, the 230 °C/2 h cured n-LTPI films did not showed obvious changes in glass transition temperature at TMA curves at <350 °C, implying low thermal expansion behavior in a broad temperature range. Figure 9 depicts the TGA curves of the 230 °C/2 h cured n-LTPI films with different IMZ loadings. By increasing IMZ loadings from 1.0 wt.% to 5.0 wt.%, the 230 °C/2 h cured n-LTPI films showed an apparent improvement on thermal decomposition behavior. The temperature at 5% original weight loss (Td5) increased from 368.1 °C for n-LTPI-IMZ-1.0 to 486.8 °C for n-LTPI-IMZ-5.0, about 55.1–173.8 °C higher than that of n-LTPI-0 (313.0 °C), indicating that the heteroaromatic bases have resulted in the inter-polymer chain interaction and the crosslinked network in the thermally cured polymer aggregates.

The dielectric constant at 10 GHz of the 230 °C/2 h cured n-LTPI films with different IMZ loadings of 1.0% to 5.0% were measured at 2.8–2.9; the dielectric loss factors at 10 GHz were measured in the range of 0.0048–0.0058, demonstrating that the addition of heteroaromatic bases in n-LTPIs did not deteriorate the dielectric properties of the thermally cured polymer films at high frequency.

## 4. Conclusions

Low temperature curable negative-tone photosensitive polyimides have been prepared by dissolving photo-crosslinkable poly (amic ester) (pc-PAE) resin, photoinitiator, photo-crosslinker, and heteroaromatic base as curing catalysts and other additives in organic solvents. The pc-PAE resin was synthesized by polycondensation of aromatic diacid chloride and diester of 2-ethoxymathacrylate, aromatic diamines in aprotic solvents. The n-LTPI viscous solution, with the addition of imidazole (IMZ) as a curing catalyst, produced high-quality photo-patterns with line and via resolution of 5–8 μm at 5–10 μm film thickness. The photo-patterned polymer film (n-LTPI-IMZ-2.0) that was thermally cured at 230 °C/2 h in nitrogen exhibited great combined mechanical and thermal properties, including mechanical properties with tensile strength as high as 189.0 MPa, tensile modulus of 3.7 GPa and elongation at breakage of 59.2%, as well as glass transition temperatures of 282.0 °C, CTE of 48.3 ppm/^◦^C, T_5_ of 426.3 °C, dielectric constant (10 GHz) of 2.8, and dielectric loss factors (10 GHz) of 0.0056, showing great potential for applications in advanced microelectronic packaging.

## Figures and Tables

**Table 1:** Lithographic performance of the low-temperature curable photosensitive polyimides.

Lithography Process	n-LTPI-0	n-LTPI- DMP-2.0	n-LTPI- QL-2.0	n-LTPI- IQL-2.0	n-LTPI- IMZ-2.0	n-LTPI- BZI-2.0
Film thickness after soft-bake/μm	9.8	9.9	9.8	10.0	9.8	9.9
Exposure energy/mJ	400~600	600~800	400~600	300~600	600~800	300~600
Film thickness after Development/μm	8.9	8.2	9.0	9.0	8.4	9.0
Film retention rate/%	90.8	82.8	91.8	90.0	85.7	90.9
Cured film thickness/μm	4.8	4.9	5.0	4.9	4.7	4.4
Resolution (Via)/μm	10	6	8	8	5	8

**Table 2:** Mechanical properties of the thermally cured n-LTPI films at 230 °C/2 h with different heteroaromatic bases as curing catalysts.

Sample	ID (%)	Elongation (%)	Tensile Strength (MPa)	Modulus (GPa)
n-LTPI-0	95.1	59.1	180.7	3.7
n-LTPI-DMP-2.0	98.0	45.2	178.1	3.8
n-LTPI-QL-2.0	98.1	19.2	113.4	3.9
n-LTPI-IQL-2.0	98.3	21.3	121.2	4.0
n-LTPI-IMZ-2.0	100.0	59.2	189.0	3.7
n-LTPI-BZI-2.0	97.1	42.0	165.3	3.7

**Table 3:** Mechanical properties of the thermally cured n-LTPI films at 230 °C/2 h with different loadings of heteroaromatic bases.

Sample	ID (%)	Elongation (%)	Tensile Strength (MPa)	Modulus (GPa)
n-LTPI-0	95.1	59.1	180.7	3.7
n-LTPI-IMZ-1.0	99.2	61.2	190.2	3.7
n-LTPI-IMZ-2.0	100.0	59.2	189.0	3.7
n-LTPI-IMZ-3.0	100.0	55.4	178.9	3.8
n-LTPI-IMZ-5.0	100.0	36.1	156.8	3.7

**Table 4:** Thermal and electrical properties of the thermally cured n-LTPI films at 230 °C/2 h with different loadings of heteroaromatic bases.

Sample	Thermal Properties			Electrical Properties	
	T_g_ (°C)	CTE (ppm/°C)	T_5_ (°C)	Dielectric Constant (10 GHz)	Dielectric Loss Factors (10 GHz)
n-LTPI-0	257.7	52.4	313.0	2.9	0.0051
n-LTPI-IMZ-1.0	274.5	49.2	368.1	2.8	0.0058
n-LTPI-IMZ-2.0	282.0	48.3	426.3	2.8	0.0056
n-LTPI-IMZ-3.0	276.4	49.2	439.8	2.9	0.0053
n-LTPI-IMZ-5.0	296.4	47.3	486.8	2.9	0.0048

## Data Availability

The data presented in this study are available on request from the corresponding author.

## References

[1] Ahne H.R., Leuschner R., Rubner R. (2010). Recent advances in photosensitive polyimides. Polym. Adv. Technol..

[2] Seino H., Haba O., Ueda M., Mochizuki A. (1999). Photosensitive polyimide-precursor based on polyisoimide: Dimensionally stable polyimide with a low dielectric constant. Polymer.

[3] Higashihara T., Saito Y., Mizoguchi K., Ueda M. (2013). Recent progress in negative-working photosensitive and thermally stable polymers. React. Funct. Polym..

[4] Fukukawa K.-I., Ueda M. (2008). Recent Progress of Photosensitive Polyimides. Polym. J..

[5] Rubner R. (1990). Photoreactive polymers for electronics. Adv. Mater..

[6] Yoda N., Hiramoto H. (1984). New Photosensitive High Temperature Polymers for Electronic Applications. J. Macromol. Sci. Part A Chem..

[7] Hou H.Q., Jiang J.G., Ding M.X. (1999). Ester-type precursor of polyimide and photosensitivity. Eur. Polym. J..

[8] Volksen W., Yoon D.Y., Hedrick J.L., Hofer D. (2011). Chemistry and Characterization Of Polyimides Derived from Poly(Amic Alkyl Esters). MRS Online Proc. Libr..

[9] Windrich F., Malanin M., Eichhorn K.J., Voit B., Lang K.D. (2014). Low-Temperature Photosensitive Polyimide Processing for Use in 3D Integration Technologies. MRS Online Proc. Libr..

[10] Jin X.Z., Ishii H. (2010). A novel positive-type photosensitive polyimide based on soluble block copolyimide showing low dielectric constant with a low-temperature curing process. J. Appl. Polym. Sci..

[11] Yuba T., Suwa M., Fujita Y., Tomikawa M., Ohbayashi G. (2002). A Novel Positive Working Photosensitive Polyimide for Wafer-level CSP Packages. J. Photopolym. Sci. Technol..

[12] Metz S., Jiguet S., Bertsch A., Renaud P. (2004). Polyimide and SU-8 microfluidic devices manufactured by heat-depolymerizable sacrificial material technique. Lab Chip.

[13] Stoffel N.C., Kramer E.J., Volksen W., Russell T.P. (1993). Solvent and isomer effects on the imidization of pyromellitic dianhydride-oxydianiline-based poly(amic ethyl ester)s. Polymer.

[14] Echigo Y., Iwaya Y., Tomioka I., Yamada H. (1995). Solvent Effects in Thermal Curing of Poly(4,4′-oxybis(phenylenepyromellitamic acid)). Macromolecules.

[15] Shin T.J., Lee B., Youn H.S., Lee K.-B., Ree M. (2001). Time-Resolved Synchrotron X-ray Diffraction and Infrared Spectroscopic Studies of Imidization and Structural Evolution in a Microscaled Film of PMDA-3,4′-ODA Poly(amic acid). Langmuir.

[16] Chen X.J., Yang J., Zhao J. (2018). The effect of solvent to the kinetics of imidization of poly(amic acid). Polymer.

[17] Matsuoka Y., Yokota K., Ogitani S., Ikeda A., Takahashi H., Ai H. (1992). Ester-type photosensitive polyimide precursor with low thermal expansion coefficient. Polym. Eng. Sci..

[18] Kotera M., Samyul B., Araie K., Sugioka Y., Nishino T., Maji S., Noda M., Senoo K., Koganezawa T., Hirosawa I. (2013). Microstructures of BPDA-PPD polyimide thin films with different thicknesses. Polymer.

[19] Li C., Wang Y., Yin Y., Li Y., Li J., Sun D., Lu J., Zhang G., Sun R. (2021). A comprehensive study of pyrazine-contained and low-temperature curable polyimide. Polymer.

[20] Brekner M.-J., Feger C. (1987). Curing studies of a polyimide precursor. J. Polym. Sci. Part A Polym. Chem..

[21] Shin T.J., Ree M. (2005). In Situ Infrared Spectroscopy Study on Imidization Reaction and Imidization-induced Refractive Index and Thickness Variations in Microscale Thin Films of a Poly(amic ester). Langmuir.

[22] Nelson A., Guerra G., Williams D.J., Karasz F.E., MacKnight W.J. (2010). Catalytic activity of benzimidazole in the imidization of polyamic acids. J. Appl. Polym. Sci..

[23] Yoon J.-Y., Jeong S., Lee S.S., Kim Y.H., Ka J.-W., Yi M.H., Jang K.-S. (2013). Enhanced Performance of Solution-Processed Organic Thin-Film Transistors with a Low-Temperature-Annealed Alumina Interlayer between the Polyimide Gate Insulator and the Semiconductor. ACS Appl. Mater. Interfaces.

[24] Ahn T., Choi Y., Jung H.M., Yi M. (2009). Fully aromatic polyimide gate insulators with low temperature processability for pentacene organic thin-film transistors. Org. Electron..

[25] Huang C., Li J., Sun D., Xuan R., Sui Y., Li T., Shang L., Zhang G., Sun R., Wong C.P. (2020). Comprehensive properties study of low-temperature imidized polyimide with curing accelerators. J. Mater. Chem. C.

[26] Sui Y., Li J., Wang T., Sun D., Huang C., Zhang F., Shan L., Niu F., Zhang G., Sun R. (2021). Low temperature curing polyimides with covalent-boned 5-aminobenzimidazole. Polymer.

[27] Unsal E., Cakmak M. (2013). Real-Time Characterization of Physical Changes in Polyimide Film Formation: From Casting to Imidization. Macromolecules.

[28] Oba M. (1996). Effect of curing accelerators on thermal imidization of polyamic acids at low temperature. J. Polym. Sci. Part A Polym. Chem..

[29] Nguyen L.T.T., Nguyen H.N., La T.H.T. (2007). Synthesis and characterization of a photosensitive polyimide precursor and its photocuring behavior for lithography applications. Opt. Mater..

[30] Li W.S., Shen Z.X., Zheng J.Z., Tang S.H. (1998). FT-IR Study of the Imidization Process of Photosensitive Polyimide PMDA/ODA. Appl. Spectrosc..

[31] Fu M.-C., Higashihara T., Ueda M. (2018). Recent progress in thermally stable and photosensitive polymers. Polym. J..

[32] Windrich F., Kappert E.J., Malanin M., Eichhorn K.-J., Häuβler L., Benes N.E., Voit B. (2016). In-situ imidization analysis in microscale thin films of an ester-type photosensitive polyimide for microelectronic packaging applications. Eur. Polym. J..

[33] Zhai Y., Yang Q., Zhu R., Gu Y. (2008). The study on imidization degree of polyamic acid in solution and ordering degree of its polyimide film. J. Mater. Sci..

